# Crosstalk between cysteine and lysine modifications: Integrating redox and metabolic regulation

**DOI:** 10.1016/j.redox.2026.104357

**Published:** 2026-08-22

**Authors:** Emily C. Mitchem, James R. Roede, Kristofer S. Fritz

**Affiliations:** Skaggs School of Pharmacy and Pharmaceutical Sciences, University of Colorado Anschutz Medical Campus, Aurora, CO, 80045, USA

## Abstract

Protein post-translational modifications (PTMs) on amino acid residues enable dynamic cellular responses to changes in metabolic and redox state. Cysteine and lysine are among the most extensively modified amino acid residues, with both undergoing a diversity of acylation and oxidative modifications. Indeed, proximal (<10 Å) cysteine and lysine residues may form integration nodes for crosstalk between metabolism and redox homeostasis pathways. This review highlights the interaction of proximal Cys-Lys residues, including influence on residue pKa by local electrostatics, cysteine-to-lysine transfer of PTM moieties, and covalent crosslinking. We discuss candidate Cys-Lys regulatory pairs in proteins involved in redox regulation, proteostasis, metabolic adaptation and inflammation. We further utilize computational modeling to identify proximity between cysteine and lysine residues in proteins known to be regulated by acylation and oxidative PTMs, and to demonstrate changes in these distances and local electrostatic potential due to lysine acetylation. Finally, we review how mass spectrometry-based proteomics and machine-learning PTM predictive tools can enable the identification, validation, and interpretation of proximal Cys-Lys interactions that regulate cellular responses to oxidative challenge and metabolic flux.

## 1 Introduction

Post-translational modifications (PTMs) enable dynamic cellular responses to environmental cues. The propensity for an amino acid residue to be modified is influenced by its microenvironment and the PTM status of proximal residues, among other factors. Indeed, lysine and cysteine residues undergo a myriad of regulatory modifications, and ongoing research is uncovering new ways in which crosstalk between these residues influences protein activity, structure, and signaling. Acylation (acetylation, propionylation, succinylation, etc.) of the lysine ε-amino group occurs enzymatically and nonenzymatically, with acyl-CoAs typically acting as the donors of the acyl groups. Acyl-CoAs are generated during metabolism of glucose, fatty acids, amino acids and other energy sources, enabling an intrinsic link between lysine acylation and metabolism. For example, acetylation of histone and non-histone proteins increases with ethanol consumption, a result of increased acetyl-CoA production [[1], [2], [3]]. Acylation can also occur on cysteine residues; where a highly nucleophilic thiol group makes them susceptible to a wide breadth of modifications. Indeed, cysteines are particularly prone to oxidative modifications by reactive oxygen and nitrogen species. While irreversible oxidative damage to cysteine residues can occur, reversible oxidative modifications like S-glutathionylation can guard cysteines from hyperoxidation. Lower oxidation state cysteine PTMs, like sulfenic acid and disulfide formation, can function as sensors of cellular redox status and are essential for many signaling pathways [4,5]. The crosstalk between the modification of cysteine and lysine residues, therefore, links thiol redox and post-translational metabolic regulation [6].

PTMs alter the electrostatic microenvironment of amino acid residues, altering their interaction with neighboring amino acids and consequentially influencing the reactivity of nearby functional groups. Therefore, along with directly affecting protein function, a PTM can create downstream functional consequences through its influence on the PTM status of neighboring residues. This review discusses recent evidence regarding cysteine and lysine modifications, the implications of their electrostatic interactions and potential consequences in cellular processes and diseases. Here, we review proximal (<10 Å) cysteine-lysine (Cys-Lys) pairs in proteins known to be regulated by PTMs on one or both Cys-Lys residues in a proximal pair. Progress in characterizing amino acid and PTM crosstalk has been aided by recent advances in proteomic methods and protein structure modeling using artificial intelligence (AI) [7,8]. Utilization of these tools for investigation into lysine acylation and cysteine redox PTMs provides a promising avenue for deciphering complex mechanisms of protein dysregulation, which are critically involved in metabolic and oxidative stress pathways central to numerous diseases and healthspan. Overall, here we review concepts surrounding how lysine may alter cysteine reactivity and localized microenvironments, how cysteine can promote lysine acetylation, and we describe several novel Cys-Lys covalent switches.

## 2 Regulatory nature of reactive oxygen species and thiol redox

Reactive oxygen species (ROS) act as both etiological factors and cellular response signal molecules in many human diseases. For instance, they are involved in the progression of neurodegeneration and cancer, as well as the development of fibrosis and inflammation in alcohol associated liver disease (ALD) and chronic kidney disease (CKD). Sulfur thiol groups, including those on small molecules like glutathione (GSH) and the side chain of cysteine residues, are widely utilized for antioxidant capacity and participate in redox signaling. Thiols may be reversibly oxidized under stress conditions by disulfide formation with GSH and Coenzyme A (CoA) to prevent irreversible modifications and permanent damage, as well as to dynamically signal cellular response to stress. Furthermore, protein activity can be regulated by thiol redox through modifications of cysteine residues involved in several key functions like structural disulfide bonds, enzyme catalysis, and metal coordination (e.g. iron sulfur clusters and ZZ domains).

Many cellular pathways essential for both internal homeostasis and response to external stimuli are redox sensitive. These processes include inflammation, T-cell differentiation, apoptosis, and insulin signaling. Redox-signaling pathways involve thiol switches (reactive cysteine residues) that can activate or inhibit components of the pathway upon oxidative modification by ROS [4]. Multiple modes of regulation are often integrated within a single pathway, and even within a single protein. Lysine residues can also be impacted by oxidative stress, particularly through adduct formation with electrophiles. Lipid peroxidation, initiated by hydrogen abstraction of a lipid by a free radical species, creates a free radical chain reaction that produces reactive aldehydes like 4-hydroxynonenol (4-HNE), and malondialdehyde (MDA), which can adduct to lysine residues [9,10]. This mechanism of disrupted protein function is particularly relevant during increased oxidative stress in metabolic disorders that induce lipid accumulation. 4-HNE adduction can also cause the formation of lysine-lysine or lysine-cysteine crosslinks, which contribute to neurodegeneration [11] and atherosclerosis [12].

The cellular effects of ROS depend on species, flux, localization, and the surrounding redox environment; controlled H_2_O_2_ signaling reversibly modifies reactive cysteines, whereas excessive oxidant exposure can lead to nonspecific cellular damage. This highlights the importance of the impact of redox signaling in response to the low rates of ROS production that occur under normal physiological conditions, compared with responses to high ROS production during stress. Here we can see the dynamic maintenance of redox homeostasis and the pleiotropic nature of redox changes, as the difference between oxidative damage and beneficial signaling is dependent upon the concentration of ROS and the microenvironment upon which it reacts [13].

## 3 Post-translational modifications and metabolic regulation

Acyl-CoAs, formed by the combination of coenzyme A and a diversity of acyl groups, are essential components of metabolism and signaling. They are generated and consumed during the citric acid cycle, fatty acid oxidation, and lipogenesis. Acyl-CoAs are also the main donors of acyl moieties for lysine acylation, making them a key link between cellular metabolism and protein regulation by PTMs. Lysine acetylation by acetyl-CoA can occur enzymatically in the nucleus and cytosol by acetyltransferases including p300 and CREBBP. However, acetylation, especially in the mitochondria, can also be non-enzymatic. The slightly alkaline microenvironment of the mitochondria favors lysine acetylation as deprotonation of the lysine amine promotes its nucleophilic attack on an acetyl carbonyl. Indeed, many lysine acetyltransferases contain an active site acidic residue to catalyze the deprotonation step in lysine acetylation [14]. Work by Carrico et al. [15] has described a semi-enzymatic lysine acylation where lysine residues near CoA binding sites can become preferentially acylated by acyl-CoAs. Proteins containing CoA binding pockets were found more likely to be acylated, and for these proteins, residues within 5 Å of the CoA sulfur in the CoA binding pocket were more likely to be modified than distal lysine residues. Non-enzymatically acetylated lysine sites are also tightly regulated through their enzymatic removal by histone deacetylases (HDACs). Sirtuins, NAD^+^ -dependent class III HDACs, regulate cell processes through their deacylation of both histones and non-histone proteins. These non-histone proteins include critical cellular targets like SOD2, NF-kB, HSP90, and p53. Interestingly, HDACs themselves can also be regulated by oxidative stress, and cysteine redox switches have been identified in class I, II and III HDACs [[16], [17], [18]].

Besides acetylation, lysine residues can be modified by many other acyl groups, providing a breadth of alterations in the charge, polarity, and size of the residue. For example, while acetylation is a 42 Da addition to a lysine which neutralizes the positively charged amine, succinylation (100 Da) adds a negative charge, and myristoylation is a large 210 Da 14-carbon chain modification. There are also acyl transferases, like the ZDHHC family that catalyze the enzymatic addition of 14-22 carbon acyl groups to amino acids including lysine and cysteine. For example, ZDHHC13 is a palmitoyl transferase that modifies cysteine residues. ZDHHC13 deficiency in mice causes liver damage, attenuates lipid accumulation and hypermetabolism, and its knock-down in Hepa1-6 cells caused mitochondrial dysfunction and ROS production [19]. Recent work also evaluates the role of cysteine S-acetylation, which was found enriched in metabolic pathways [20]. Specifically, this study identified cysteine PTM regulation on GAPDH, an enzyme central to glycolysis. S-acetylation was found on murine GAPDH at Cys150 (homologous to Cys152 on human GAPDH), a catalytic cysteine essential for the conversion of glyceraldehyde-3-phosphate to 1,3-bisphosphoglycerate. Cys150 acetylation inhibited GAPDH catalytic activity, which also occurs by hyper-oxidation [21] of this residue. GAPDH is also inhibited by CoAlation at Cys23 and Cys245 [22]. Cysteine S-CoAlation is induced by acetyl-CoA under oxidative or metabolic stress and protects protein thiols from overoxidation [23]. Other metabolic enzymes also regulated by thiol redox include pyruvate kinase (PKM2) which catalyzes the last step in glycolysis, and G6PDH, responsible for the first step in the pentose phosphate pathway [24].

## 4 Thiol redox and lysine acylation crosstalk

The pathways for sensing cellular metabolic and oxidative states may be further connected through crosstalk between coordinated sets of cysteine and lysine residues. James et al. have described the influence of proximal cysteines on acetylation of lysines via the non-enzymatic transfer of an acetyl moiety from a cysteine to a nearby lysine [6,25]. This interaction is more favorable than direct N-acetylation of lysine residues due to the higher nucleophilicity of cysteine thiols compared to lysine ε-amines, allowing for enhanced nucleophilic attack on the acetyl carbonyl. Additionally, lysine residues can affect the reactivity of proximal cysteine residues through their impact on cysteine pKa. The deprotonation of a cysteine thiol to an anionic thiolate is typically not favored at physiologic pH; however, interaction with a nearby positive charge can stabilize the negatively charged thiolate state, thereby depressing cysteine pKa. The increased electron density in the thiolate form enhances the nucleophilicity, and therefore reactivity, of cysteine residues. Proteomic analysis of murine hepatic tissue revealed that proximity (<15 Å) between a cysteine and lysine increased the likelihood of redox modification on the cysteine to occur from alcohol metabolism [26]. Moreover, sequence analysis of the cysteine proteome revealed lysine residues to be an overrepresented amino acid within 6 residues upstream and downstream of modified cysteine residues [27]. They calculated a 55.8% increase in lysine residues within the 13 amino acid windows around a sulfenated cysteine compared to the lysine occurrence expected by chance. Lysine was also overrepresented, but to a lesser extent, around nitrosylated, glutathionylated, and acylated cysteines. Work by Pehar et al. [28] further supports the hypothesis of an interaction between S-glutathionylation and lysine acetylation. They show that a decrease in glutathione levels via knockout of glutamate-cysteine ligase (GCL) modifier subunit in astrocytes caused an increase in lysine acetylation, and importantly cysteine was found enriched near these acetylation sites [28].

Lysines and cysteines can also act in union to form a redox switch through nitrogen-oxygen-sulfur (NOS) bridges. This cross-link forms between a lysine nitrogen and one or two mono-oxidized cysteine residues (NOS and SONOS bridges respectively). Rabe von P et al. [29] performed a PDB protein crystal structure data search and identified 266 structures with electron density maps and Cys-Lys proximity suggesting formation of these bridges. They proposed that in response to oxidative stress or oxygen availability Cys-Lys bridges may act as allosteric regulators through their impact on structure, or as reversible modulators of activity when catalytic lysine or cysteine residues are involved. Computational modeling showed H_2_O_2_ and •OH to be capable of exergonically oxidizing cysteine and lysine residues to form these bridges. NOS bridge formation is predicted to occur via a thio(hydro)peroxy acid intermediate, followed by homolytic dissociation. The resulting hydroxyl radical can generate a radical on the nitrogen of a nearby lysine, which combines with the oxygen radical on the oxidized cysteine residue to form a NOS bridge. NOS bridges were also suggested to protect from irreversible oxidation of cysteines by preserving the cysteine in a mono-oxidized state.

The following sections discuss Cys-Lys pairs in proteins known to be regulated by thiol redox and/or lysine acylation. Interacting residues are identified based on a proximity of <10 Å in 3D space between the cysteine sulfur and lysine ε-amine using protein structures published in Protein Data Bank (RCSB.org). The 10 Å cutoff was chosen based on an analysis by James et al. of acetylated-lysine to cysteine distance in structural models of proteins based on a dataset of N-acetylated mouse liver peptides [25]. They found increased N-acetylation when a lysine residue was near a cysteine residue; this increase was most significant at 2.5-5Å and decayed to background at distances >10 Å. This cutoff is also based on predictions of spheres of interaction of protein residues; however, a few interactions have been identified in the 10-15 Å range. It is important to note that the distances monitored in these structures are dependent on interaction with other molecules, e.g. distances may drastically change in a protein that is bound to ligands, activity modulators, nucleotides, or other proteins. The protein structures and bound ligands used for cysteine-lysine distance monitoring are noted in Table 1. Acetylation neutralizes the lysine ε-amino charge and may alter local electrostatic interactions and residue proximity. The reactivity and PTM profiles of important cysteine and lysine residues for each reviewed protein example will also be discussed. We provide these examples as structurally plausible targets of regulatory Cys-Lys interaction, and as a foundation toward consideration of PTM crosstalk in the interpretation of protein regulation. The proteins discussed below are prominently involved in neurodegeneration, cancer, and metabolic diseases and demonstrate the cysteine thiol to lysine Nε-acylation proximity as a strategy for the mechanistic understanding of protein regulation which may lead to the identification of therapeutic targets for myriad diseases.

**Table 1:** Protein structures used for residue distance and ESP modeling.

Protein Name	Uniprot Accession ID	PBD ID	Method	Resolution (Å)	Residue Range Included	Bound Proteins and Ligands
KEAP1	Q14145	8WFG	Xray	2.25	N49-L179	
PRDX1	Q06830	4XCS	Xray	2.1	M1-P179	homodimer, glycerol, CHAPS
DJ-1	Q99497	7PA2	Xray	1.21	A2-D189	8RK64
TDP-43	Q13148	4Y0F	Xray	2.65	T103-S180	homodimer, ssDNA
P62	Q13501	6TGY	EM	3.5	A2-C105	
PPAR-γ	P37231	3E00	Xray	3.1	M107-Y477	RXRα, DNA, GW9662, 9-cis Retinoic Acid and NCOA2 Peptide
HMGB1	P09429	2LY4	NMR	N/A	G2-E84	p53

We describe a model in which Cys-Lys pairs function through several non-mutually exclusive mechanisms: lysine residues can tune cysteine pKa and thiolate formation; cysteine residues can act as transient acyl acceptors that promote lysine acylation; redox-sensitive Cys-Lys pairs form covalent NOS or SONOS bridges; and modifications of either residue may alter regional electrostatics, sterics, or conformational dynamics. The following sections highlight representative proteins in which these mechanisms may contribute to redox regulation, proteostasis, metabolic adaptation, and inflammatory signaling.

## 5 ***Cys-Lys crosstalk in redox regulation, proteostasis, metabolism, and inflammation***

### 5.1 Redox regulation (KEAP1, PRDX1, and DJ-1)

The proteins described in this section utilize thiol switches to maintain a homeostatic cellular redox balance through the control of antioxidant gene transcription or through the direct management of ROS. The amino acid microenvironment is critical to the reactivity of these thiol switches, and we identify lysine residues proximal to these essential cysteine residues, the acetylation of which may influence the activity of these proteins.

#### 5.1.1 KEAP1

**Fig. 1:**
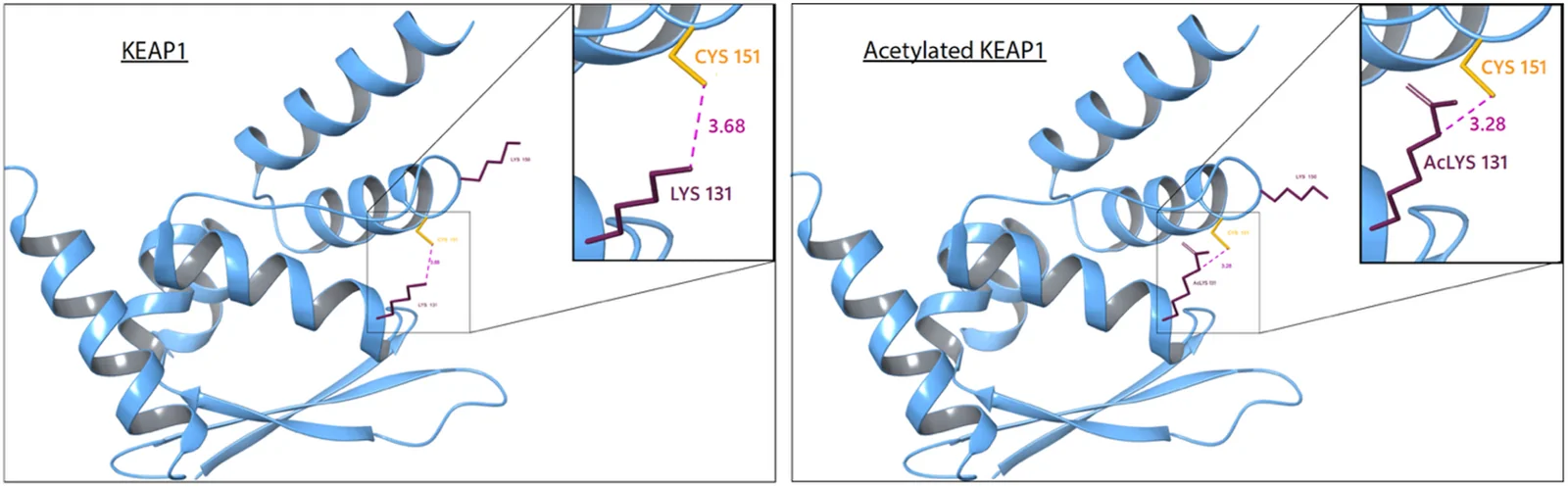
Cys151-Lys131 Pair in KEAP1 Key cysteine residues in KEAP1 function as thiol sensors regulating KEAP1's mediation of the interaction between Nrf2 and the ubiquitin ligase Cul3. Among these residues is Cys151 in the BTB domain of KEAP1, this cysteine residue is located only 3.68 Å from Lys131. Acetylation of Lys131 may influence the reactivity of Cys151 to electrophiles that activate KEAP1/Nrf2 signaling. The structure of the BTB domain (aa 49-179) is shown with cysteine residues of interest depicted in yellow, lysine residues of interest in maroon and all other residues as light blue.

Cys-Lys interactions may be at play in the regulation of Kelch-like ECH associated protein 1 (KEAP1), a redox signaling protein that responds to ROS and electrophiles. The KEAP1/Nrf2 pathway is involved in cancer, neurodegenerative diseases, atherosclerosis, and diabetes through control of antioxidant pathways in response to xenobiotics and oxidative damage. In the absence of stress, KEAP1 represses Nrf2 by facilitating its interaction with Cul3. The polyubiquitination of Nrf2 by Cul3 marks it for proteasomal degradation, preventing Nrf2 translocation to the nucleus where it acts as a transcription factor for antioxidant response elements (AREs), thereby regulating transcription of NQO1, PRDX1, GSH S-transferases, and other detoxifying enzymes. The activation of KEAP1 is highly regulated by thiol sensors. Dinkova-Kostova et al. [30] found that 5 of the 25 cysteine residues in KEAP1 are particularly reactive to the electrophile dexamethasone 21-mesylate: Cys257, Cys273, Cys288, Cys297, and Cys613. Remarkably four out of five of these cysteines are within 3 amino acids of a lysine; 273 is the exception and is adjacent to an arginine. Further work into the cysteine regulation of KEAP1 has differentiated the cysteine residues by inducibility to electrophile or oxidant stressors. Cys151, Cys273, and Cys288 function as sensors in KEAP1 by acting as receptors in Michael addition reactions with electrophiles. Two of these critical regulatory cysteine residues are proximal to a lysine- Cys151 is 3.68 Å from Lys131 and Cys288 is next to Lys287 (Fig. 1).

Intriguingly, Lys131 has been found acetylated in HeLa and Jurkat cells [31,32]. Lys131 of KEAP1 is also succinylated, causing Nrf2 activation [33]. The presence of lysine residues near many of KEAP1's regulatory cysteines may be due to the depression of cysteine pKa when it is near a positively charged lysine, increasing the propensity for thiolate formation. Thiolates are more nucleophilic than thiols, making them generally more reactive to electrophiles and to oxidants like H_2_O_2_, allowing them to act as redox switches of KEAP1 [34]. Due to the dualistic role of KEAP1/NRF2 signaling in many diseases it is of high importance to evaluate the impact of acylation on the lysine residues proximal to KEAP1's key redox sensitive cysteines.

#### 5.1.2 PRDX1

**Fig. 2:**
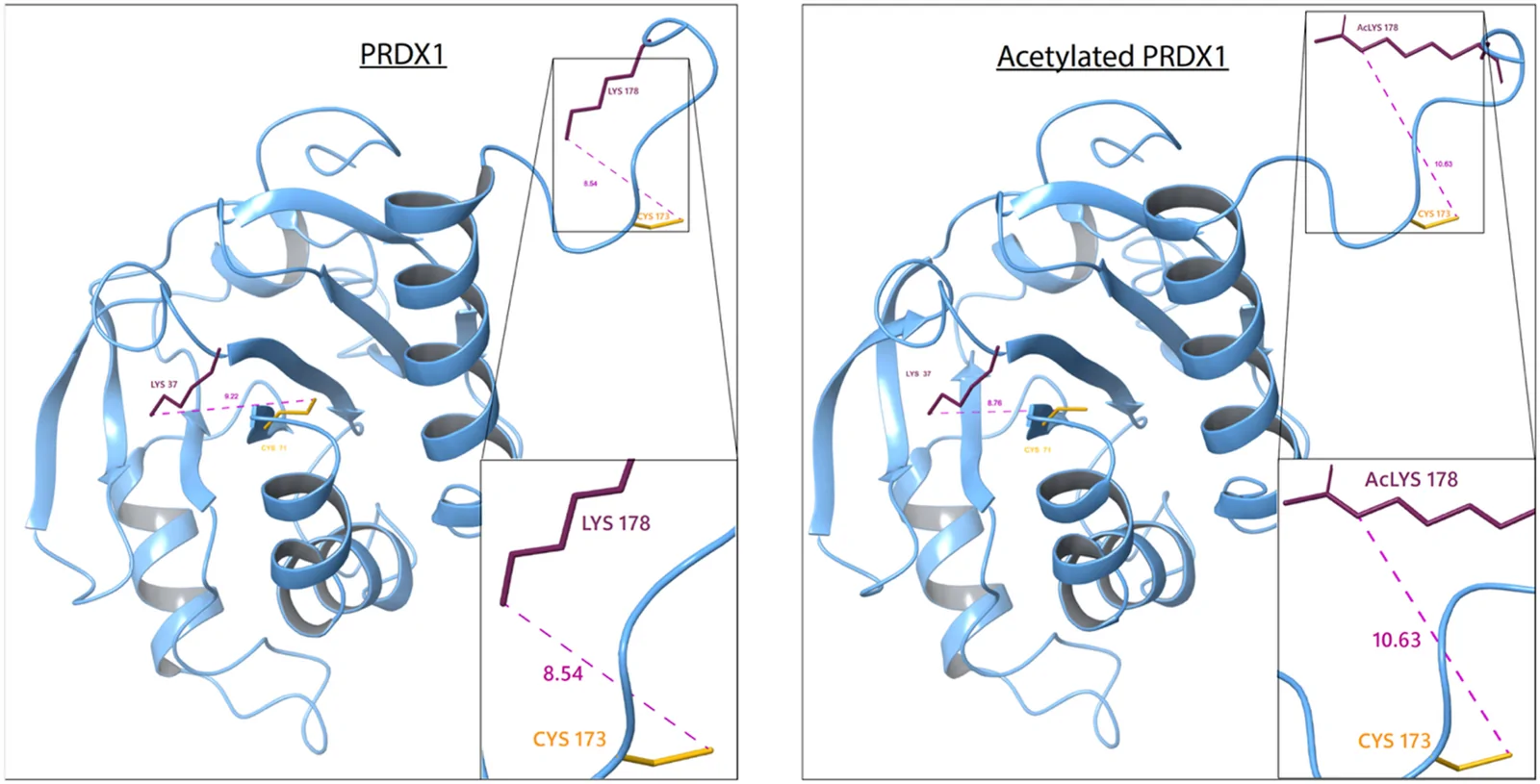
Cys173-Lys178 Pair in PRDX1 In the enzymatic reduction of peroxides by PRDX1 Cys52 is oxidized and subsequently forms a disulfide with the resolving cysteine residue, Cys173. Cys173 is proximal to Lys178, whose acetylation may influence resolving cysteine reactivity, disulfide formation, or catalytic recycling.

Peroxiredoxins (PRDXs) are antioxidant enzymes that reduce peroxides, protecting the body from ROS accumulation and ferroptosis. PRDX1 is part of the 2-cysteine class of PRDXs, with residues Cys52 and Cys173 involved in the catalytic disulfide bond formation necessary for the conversion of H_2_O_2_ to H_2_O. Cys52 is the peroxidatic residue that reacts with H_2_O_2_ to form cysteine-sulfenic acid. Cys52 then forms a disulfide with the resolving cysteine, Cys173. This disulfide is broken to reform the thiols/thiolates by thioredoxin 1 (Trx1). The participation of Cys173 in PRDX1 catalytic activity may be influenced by acetylation at nearby Lys178 (8.5 Å) which was identified in Jurkat cells [31] (Fig. 2). Indeed, lysine acetylation is directly linked to cysteine redox regulation in PRDX1 as acetylation at Lys197 (13.8 Å from Cys173 using AF3 structure) by the acetyltransferase MOF prevents PRDX1 hyperoxidation [35]. This residue is deacetylated by HDAC6, and inhibition of HDAC6 has been shown to protect cells from cell death following H_2_O_2_ treatment [36]. Additionally, PRDX1 is inhibited by deacetylation by SIRT2 at unknown residues and the overexpression of SIRT2 in breast cancer cells was found to increase PRDX1 oxidation and susceptibility to ROS induced death [37]. Unlike the efficient H_2_O_2_ scavenging enzyme catalase, PRDX1 easily becomes hyperoxidized at high concentrations of H_2_O_2_ [38]. Yet, the overoxidation of cysteines is an important feature in PRDX1's role in redox sensing, as Cys-sulfonic acid formation converts PRDX1 into an oligomeric chaperone protein [39]. Investigation into PRDX1 redox regulation via modulation of acetylation is important due to its antioxidant and chaperone function in pathways related to apoptosis, DNA damage and cancer [40,41].

#### 5.1.3 DJ-1

DJ-1 (PARK7), one of the top 50 expressed proteins in cells, is an oxidative stress sensor first identified in cancer and neurodegeneration [42], but also of importance in kidney diseases and type II diabetes. Mutations in the gene that encodes DJ-1 lead to the development of early-onset Parkinson's disease. DJ-1 is cytoprotective through regulation of oxidative stress response transcription factors (TFs) including Nrf2 and HIF-1α. DJ-1 is also involved in neural oxidative stress through synthesis and reuptake of dopamine [42], and by acting as a glyoxalase III (Glo3) to detoxify the advanced glycation end (AGE) product methylglyoxal by converting it to lactate [[43], [44], [45]]. Critical for the enzymatic functions of DJ-1 is the catalytic and redox sensing residue Cys106.

Cys106 is particularly sensitive to oxidation due to its low pKa of 5.4, arising from the stabilization of the thiolate via hydrogen bonding with a proximal protonated glutamic acid residue, Glu18 [46]. The electrostatic interaction with Glu18 promotes Cys106 oxidation to sulfinic acid, which is otherwise an unstable modification due to facile further oxidation to sulfonic acid. When the progression to sulfonic acid does occur DJ-1 is rendered inactive. Furthermore, the formation of Cys106-sulfinic acid increases DJ-1 localization to the mitochondrial matrix [47], where it affects mitochondrial morphology and mitophagy [42]. DJ-1 is also cytoprotective in response to oxidative stress through interaction with Apoptosis Signal Regulating Kinase 1 (ASK1) to prevent cell death pathways. DJ-1 suppresses dissociation of ASK1 from Trx1 in response to H_2_O_2_^48^, but mutation of Cys106 to a serine or an alanine prevents DJ-1 from interacting with ASK-1 under H_2_O_2_ treatment [48,49]. A C106DD mutant, which approximates higher cysteine oxidation states by inserting two aspartate residues at position 106, constitutively binds ASK1, indicating that Cys106 acts as a pro-survival redox sensor whereby its oxidation allows suppression of ASK1 apoptotic signaling [49]. Importantly, C106 is located near lysine residue Lys132 (11.6 Å), the acetylation of which may impact Cys106's functioning as a redox sensor (found acetylated in HeLa and Jurkat cells [31,32]). The other two cysteine residues in DJ-1, Cys46 and Cys53, are part of the dimer interface and their nitrosylation results in inactive DJ-1 [50]. The importance of dimerization is underscored by the increased risk for PD with mutations that disrupt the dimer [51]. Analysis of the structure of DJ-1 reveals Cys46 to be proximal to Lys12 (9.6 Å away).

### 5.2 Proteostasis (TDP-43 and P62)

The maintenance of protein homeostasis (proteostasis) includes the facilitation of protein folding as well as the proteolytic degradation of misfolded proteins. PTMs influence protein folding and the propensity for aggregation, with oxidative modifications being a common cause of protein misfolding. This section addresses the potential influence of cysteine-lysine proximity on protein aggregation and the activity of proteins regulating cellular proteostasis.

#### 5.2.1 TDP-43

A prominent example of the importance of proper PTM regulation and crosstalk can be seen through the dysfunction in PTM regulation of TAR DNA binding protein 43 kDa (TDP-43) which contributes to the pathogenesis of neurodegenerative diseases including Alzheimer's disease, Parkinson's disease, and amyotrophic lateral sclerosis (ALS). Functional TDP-43 is expressed primarily in the nucleus where it regulates RNA splicing and processing, and cellular stress responses. However, accumulation of TDP-43 in the cytoplasm induces toxicity through both loss of normal TDP-43 function and gain of aberrant function associated with aggregation. Cytoplasmic mislocation of TDP-43 is related to several PTMs including hyperphosphorylation, truncation, and ubiquitination.

TDP-43 aggregation is influenced both by cysteine oxidation and lysine acetylation. Evaluation of high molecular weight species in cells treated with H_2_O_2_ or arsenite, which also promotes ROS generation, showed TDP-43 to be susceptible to oxidation of cysteines into disulfides, causing the formation of intra- and inter-molecular crosslinks and protein aggregates [52]. Cys173 and Cys175, located near Lys176 (11.16 Å and 9.90 Å away respectively), were found to form a disulfide in the HMW species. Cys198, which is 13.3 Å from Lys192 using an AF3 structure, also participated in crosslink formation. Acetylation of Lys192 in RRM2 promotes accumulation of hyper-phosphorylated TDP-43 [53]. The mutation of these two lysine residues to arginine residues delayed TDP-43 aggregation following arsenite exposure, suggesting a role of lysine acetylation in oxidative stress induced crosslink formation. Moreover, TDP-43 is deacetylated by HDAC6, and HDAC6 inhibition decreases accumulation of misfolded TDP-43 [54]. Further insight into how lysine and cysteine crosstalk impacts the modification of TDP-43 and sequential aggregate formation would provide a potential intervention point in the prevention of TDP-43 proteinopathies.

#### 5.2.2 P62

**Fig. 3:**
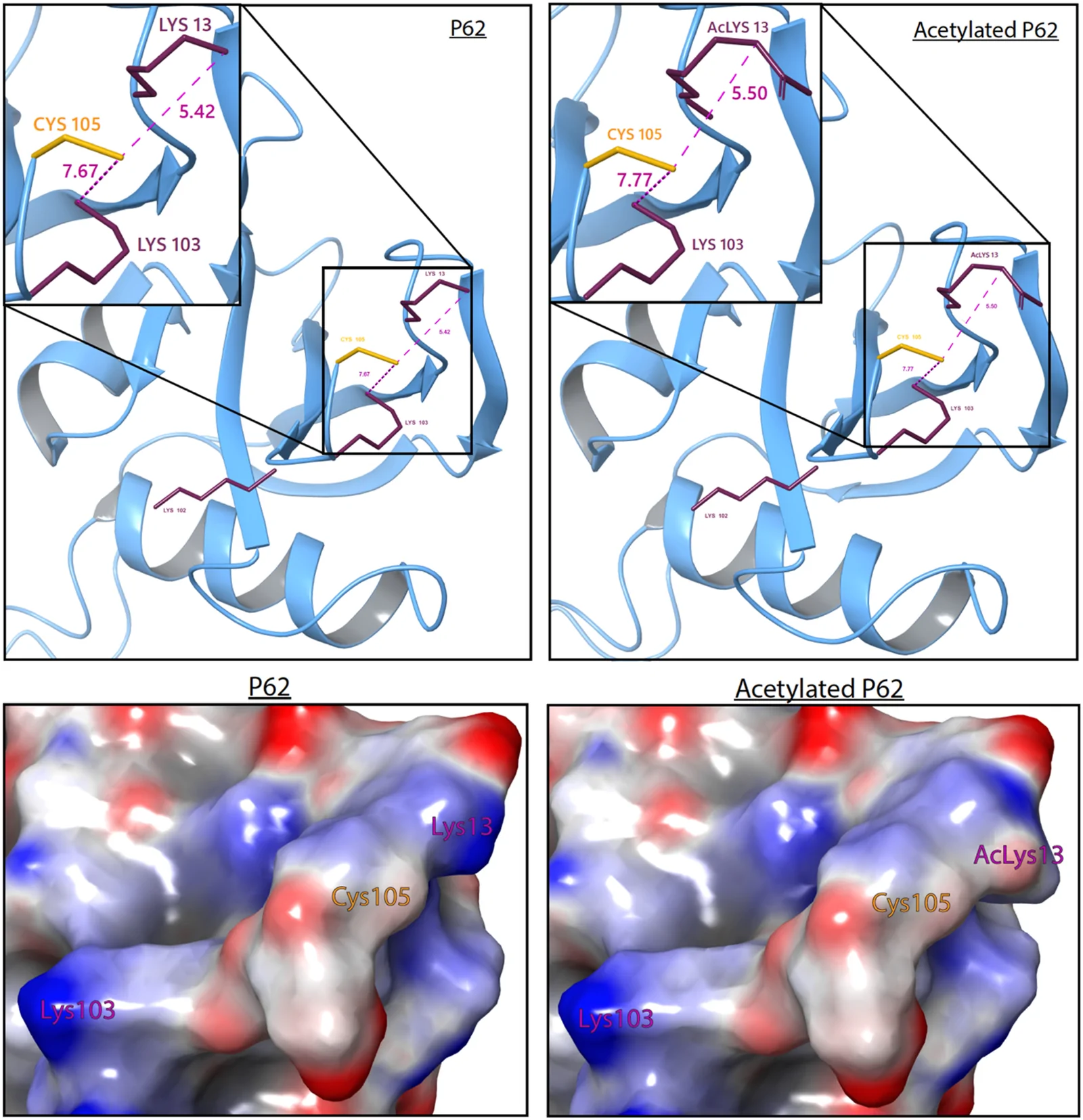
Cys-Lys Pairs in P62/SQSTM1 Cys105 and Cys133 in P62 contribute to P62 oligomerization and cargo aggregation to form inclusion bodies cleared by autophagy. Cys105 is proximal to Lys103, Lys 100, and Lys13. The acetylation of these lysine residues may impact the oxidation of Cys105, which promotes intermolecular disulfide formation to create P62 homo-oligomers. As shown in the ESP mapping of this protein, the electron density around P62 changes with acetylation of the proximal lysine residues. Red coloring of the ESP surface indicates negative potential, and blue positive potential.

P62 (SQSTM1) is an autophagy receptor that facilitates protein aggregate autophagy. It connects cargo selected for degradation to Atg8 proteins on the inner surface of the budding membranes that become autophagosomes. P62 is involved in inflammation through several pathways including inflammasome formation, NF-κB and Nrf2 signaling. The inflammasome pathway is downregulated by p62-dependent degradation of its components [40]; however, with sustained stress p62 can form P-bodies activating NLRP3 inflammasome assembly [55]. The accumulation of p62 inclusion bodies also occurs with age and neurodegenerative disorders and is a sign of proteostasis dysfunction. P62 disulfide linked conjugates and aggregates are formed in HeLa cells after administration of H_2_O_2_ directly, or of PR-619, a redox cycler producing H_2_O_2_^37^. Cys105 in the PB1 domain was determined to be necessary for the increase in formation of p62 aggregates in response to ROS. Further, the absence of these cysteines decreased autophagy and cell survival under oxidative stress. Notably, Cys105 is just downstream of several lysine residues: Lys100, Lys102, and Lys103 (10.1 Å, 15.6 Å, 7.7 Å, Fig. 3). In 3D space Cys105 is also proximal to Lys13 (5.4 Å) which has been found acetylated in HeLa cells [32]. Due to the multi-faceted role of P62 in inflammation, cancer, and neurodegeneration it is important to understand how P62 body formation may be regulated by interactions between redox and acyl PTMs on proximal cysteine and lysine residues.

### 5.3 Metabolic adaptation and inflammation (PPAR-γ and HMGB1)

PTMs are a crucial means of cellular response to the environment, for example, lysine acetylation is an important modulator of the activity of many metabolic enzymes, and thiol switches and ROS second messengers are integral components of signal transduction in immune pathways [56,57]. This section includes proteins involved in metabolic adaptation and inflammation whose PTM regulation may represent a nexus of lysine acylation and thiol redox signaling.

#### 5.3.1 PPAR-γ

PPAR-γ is a nuclear receptor transcription factor that is induced by lipophilic ligands to act as a regulator of lipid accumulation and adipogenesis. PPAR-γ1 and PPAR-γ2 are encoded from the same gene, but PPAR-γ2 includes an additional 28 amino acids at the N-terminus [58]. PPAR-γ2 is mainly expressed in adipose tissue, while PPAR-γ1 is more ubiquitously expressed. PPAR-γ contributes to hepatic steatosis in ALD and NAFLD, but improves insulin resistance and liver inflammation through upregulating IκBα to inhibit NF-κB. Additionally, PPAR-γ activation protects from neuronal loss and improves neurological function after traumatic brain injury by attenuating ferroptosis in neuronal cells [59]. Endogenous activators of PPAR-γ include fatty acids and eicosanoids, which act at the ligand binding domain.

**Fig. 4:**
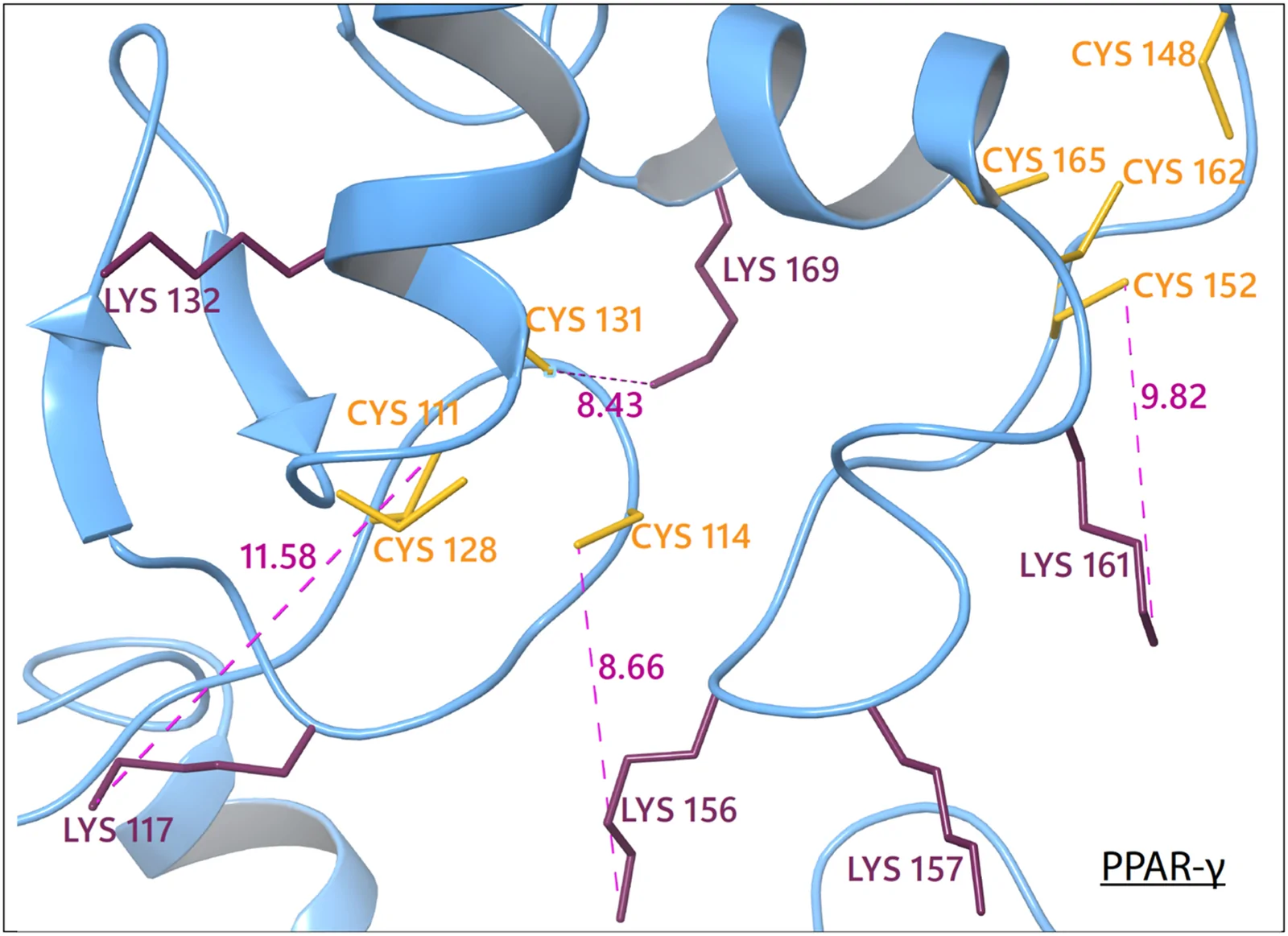
Lysine Caging of Cysteines in PPAR-γ1 Activated PPAR-γ heterodimerizes with RXR-alpha and binds to peroxisome proliferator response elements (PPREs) via two zinc finger domains in the DNA binding domain (DBD). The zinc finger domains contain two Zn^2+^ ions coordinated to 4 cysteine residues each [Cys111, 114, 128, and 131] and [Cys148, 152, 162 and 165]. These cysteine residue motifs are each surrounded by several lysine residues which suggests a potential electrostatic microenvironment that stabilizes cysteine thiolates required for zinc coordination. (Note: PPAR-γ2 includes an additional 28 amino acids at the N-term versus PPAR-γ1).

PPAR-γ activity is increased by sulfhydration at Cys139, leading to increased glucose uptake and triglyceride storage [60]. This modification of a thiol to persulfide is catalyzed by hydrogen sulfide which can be produced from cysteine by cystathionine γ lyase (CSE). Cys139 in human PPAR-γ2 corresponds to Cys111 in the conserved motif in PPAR-γ1 and might be paired with Lys117 (11.6 Å). The control of lipid metabolism by PPAR-γ is also influenced by lysine acetylation. Lys156 and Lys157 in the DNA binding domain of PPAR-γ1 were found acetylated in HEK293 cells [61]. Acetylation of these residues enhanced PPARγ-mediated upregulation of lipogenesis. Lys157 was also found to be deacetylated by SIRT1, suggesting a potential mechanism by which SIRT1 attenuates adipogenesis. These lysines are proximal to several cysteine residues (Cys148, Cys152, Cys162, and Cys165 in PPAR-γ1) suggesting PPAR-γ acetylation may occur through cysteine to lysine transfer of acetyl moieties (Fig. 4). These cysteine residues are involved in Zn^2+^ binding in PPAR-γ′s zinc finger domains which confer binding to peroxisome proliferator response elements in DNA. There is an interesting caging of these cysteines by several surrounding lysine residues, we predict that the electrostatic interactions with these lysine residues aid in the stabilization of the thiolate form of the zinc bound cysteine residues.

#### 5.3.2 HMGB1

**Fig. 5:**
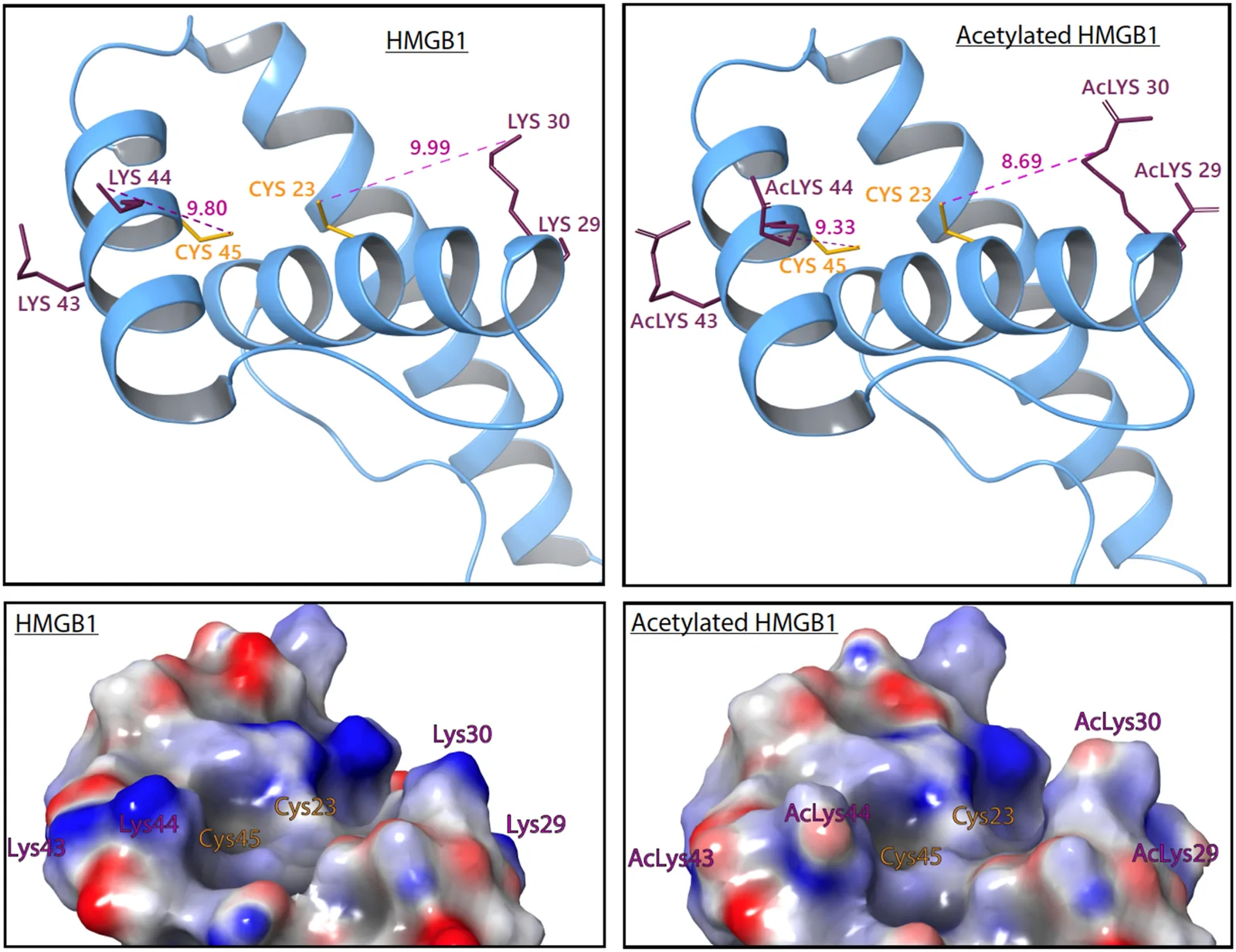
Cys-Lys Pairs in HMGB1 Immune signaling by extracellular HMGB1 is dependent upon the redox states of its cysteine residues. A Cys23 and Cys45 disulfide is required for binding of HMGB1 to the TLR4-MD2 complex and proinflammatory cytokine release, in contrast, the reduction of all 3 HMGB1 cysteines is required for HMGB1 to bind to CXCR4 and activate leukocyte chemotaxis [69]. Cys23 and Cys45 are proximal to Lys29 and Lys30, and Lys43 and Lys44 respectively. The acetylation of these residues may alter the electrostatic environment surrounding Cys23/Cys45 and likely influences disulfide formation, redox state, and HMGB1 secretion.

High mobility group box 1 (HMGB1) is a protein with DNA chaperoning roles in the nucleus, but upon extracellular localization it acts as a damage-associated molecular pattern (DAMP), triggering the release of proinflammatory cytokines. PTMs, including those on cysteines and lysines, influence HMGB1 secretion and release. The A box of HMGB1 contains redox sensitive cysteines Cys23 and Cys45, which form a disulfide through PrxI- and PrxII-mediated H_2_O_2_ oxidation [62] that is essential for HMGB1 secretion and subsequent induction of TNF release [63]. Interestingly, both of these cysteine residues are near lysines, with Cys23 proximal to Lys29 and Lys30 (11.4 Å and 10.0 Å) and Cys45 to Lys43 and Lys44 (10.5 Å and 9.8 Å), all of which have been found acetylated *in vitro* [31,32,64] (Fig. 5). Treatment of LOVO and SW620 intestinal cells with an acetyltransferase P300 inhibitor increased the levels of acetylation on Lys29 of HMGB1, suggesting it is a target of writers and erasers of acetylation [64]. N-acetylation also impacts HMGB1 cytokine activity; acetylation of lysine residues in the NLS1 (aa 28-44) and NLS2 (aa 179-185) causes HMGB1 shuttling out of the nucleus [65]. The PTM regulation of HMGB1 has been linked to many inflammatory diseases [66] in addition to blood-brain barrier damage from cerebral venous thrombosis [67] and hypoxia/reoxygenation damage in hemorrhage shock [68]. It is therefore important to determine how the acetylation and oxidative PTMs of HMGB1 controlling its extracellular release are interrelated.

## 6 Conclusions

Proximal cysteine-lysine interactions include electrostatic interactions influencing local residue pKa, PTM moiety transfer between residues, and covalent crosslinking. The interactions between Cys-Lys pairs likely impact proteins involved in a diversity of cellular pathways and processes, and disruptions in their structural and functional modifications can impact many disease states. Some of the predominant diseases connected with cysteine and lysine PTM changes include cancer, neurodegeneration, kidney disease, and metabolic disorders. The identification of Cys-Lys pairs and investigation into the pleiotropic consequences of these interactions on protein localization, stability, and function may therefore reveal biochemical mechanisms of disease etiology and novel therapeutic targets. The knowledge of cysteine-lysine interactions can also be leveraged for the identification of target residues for drug ligand binding and crosslinking sites in biologics and peptide bioengineering. The coordinated Cys-Lys interacting pairs reviewed above describe only a representative selection among the vast potential of Cys-Lys interactions in the proteome. The identification of uncharacterized Cys-Lys pairs is likely to be aided by recent advances in proteomic analytical techniques and machine-learning based predictive tools.

The use of quantitative mass spectrometry proteomics of small molecule probes selective for distinct oxidation states [70], and the click-chemistry based approach that can identify reduced vs oxidized cysteine ratios allows for site-specific and sensitive determination of global cysteine PTM mapping [71]. Using high-speed MS/MS acquisition together with acetyl-lysine immunoprecipitation with magnetic beads for efficient acetyl-peptide enrichment Soens et al. established an expanded atlas of 17,938 mouse lysine acetylation sites [72]. Additionally, by incorporating AlphaFold2 structural predictions they found acetylation to frequently occur on lysine residues with high model confidence scores (pLDDT), indicating that in contrast to phosphorylation, which tends to occur in intrinsically disordered regions, acetylation is common in structured regions. AlphaFold (AF) and the AF database of predicted protein structures have greatly expanded structural coverage of the proteome and AF structures have been used to calculate cysteine and lysine proximity and identify Cys-Lys pairs [26]. The newest AF model, AF3, uses a denoising diffusion objective to predict raw atom coordinates; this method allows for small scale definition as well as large-scale general structure prediction [73]. AF3 structures provide accuracy of local geometry predictions (e.g. proximal amino acid residue distances) even when the position of the locally folded region in reference to distal regions of the protein is uncertain. Among other improvements over AF2, AF3 features the option of adding PTMs to the input sequence, including Lysine N-acylations and Cysteine palmitoylation, malonylation and nitrosylation. AF3 models modified protein residues at a 51% success rate (%structures with a RMSD<2 Å). PTM predictions based solely on primary structures and sequence motifs have resulted in models with low prediction accuracy, yet recently the integration of spatial and structural information into deep learning models, and the adaptation of large language models to proteomics has allowed for significant progress in PTM prediction. Indeed, protein language models, which function similarly to natural language processing but replace words for amino acid residues, have been used to predict PTMs like phosphorylation [74], succinylation [75], and acetylation [76].

In addition to PTM prediction, AI is aiding in the understanding of the functionality of these PTMs [77], the reactivity of cysteines, and the role of redox state on protein structure. Reactive cysteine residues, particularly in the context of covalent binding to ligands, are predicted by CovCysPredictor [78]. This machine learning model incorporates residue microenvironment, solvent exposure, and localization of the cysteine in a ligand pocket to identify covalently binding cysteines to be targeted for small-molecule drug binding. AIUPRED predicts IDRs which enable flexible interactions between proteins and domains, and structural changes in response to the environment [79]. In AIUPRED a transformer neural network architecture replaces the logistic regression approach utilized in the earlier IUPRED. Interestingly, the probability of the residue being part of a disordered region can be compared between a protein in a cysteine stabilized state or with a cysteine to serine mutation, demonstrating the impact of cysteine redox on protein stability and folding. The integration of these novel tools may enable further comprehension of cysteine and lysine PTM crosstalk as a nexus between metabolic and redox protein regulation. Overall, future work should combine site-directed mutagenesis, redox selective cysteine chemoproteomics, acetyl-lysine enrichment, intact protein MS, pKa prediction and functional assays to test whether modeled Cys-Lys pairs serve as redox-metabolic switches.

## 7 Computational methods

To measure distances between the Cys-Lys pairs reviewed above and visualize predicted changes in protein structure and electrostatics from lysine acetylation, the following methods were employed. X-ray crystallography, NMR, or cryo-EM structures were downloaded from RCSB PDB (Table 1). Acetylation modifications were made in Discovery Studio (v25.1.0.24284, BIOVIA, San Diego, CA) and exported as mol2 files. The acetylated and unmodified structures were input into Schrödinger Maestro Suite (release 2026-2, Schrödinger LLC, New York, NY) where proteins were prepared and minimized. For protein preparation all other ligands, proteins and solvent were removed. Then side chains were repaired, hydrogens added, and the protein was protonated at physiological pH. Bond orders were not assigned or missing amino acid sequences added. The prepared structure was then minimized using default parameters of 0.3 Å RMSD and OPLS4 forcefield. The distances between cysteine and lysine residues were measured between the cysteine's sulfur and the nitrogen of lysine's ε-amine. Electrostatic potential (ESP) surfaces were applied using a 1.4 Å probe radius. Structures from AlphaFold3 are used for distance measurements when no experimental structure was available for the region of interest, these instances are noted with an ‘AF3’ annotation.

## CRediT authorship contribution statement

**Emily C. Mitchem:** Writing – review & editing, Writing – original draft, Methodology, Formal analysis. **James R. Roede:** Writing – review & editing, Supervision, Conceptualization. **Kristofer S. Fritz:** Writing – review & editing, Supervision, Conceptualization.

## Ethics declaration

This study was conducted in accordance with the ARRIVE (Animal Research: Reporting of In Vivo Experiments) guidelines. This study was approved by the Institutional Animal Care and Use Committee.

## Funding sources

This work was funded in part through 10.13039/100000002NIH grants by ECM (NIEHS T32 ES029074), JRR (NIEHS R01 ES027593), and KSF (NIAAA R01 AA030024).

## Declaration of competing interests

The authors of the manuscript “Crosstalk Between Cysteine and Lysine Modifications: Integrating Redox and Metabolic Regulation” declare no competing interests.

## Data Availability

No data was used for the research described in the article.

## References

[1] Mews P., Egervari G., Nativio R., Sidoli S., Donahue G., Lombroso S.I., Alexander D.C., Riesche S.L., Heller E.A., Nestler E.J. (2019). Alcohol metabolism contributes to brain histone acetylation. Nature.

[2] Harris P.S., Roy S.R., Coughlan C., Orlicky D.J., Liang Y., Shearn C.T., Roede J.R., Fritz K.S. (2015). Chronic ethanol consumption induces mitochondrial protein acetylation and oxidative stress in the kidney. Redox Biol..

[3] Shepard B.D., Tuma D.J., Tuma P.L. (2010). Chronic ethanol consumption induces global hepatic protein hyperacetylation. Alcohol Clin. Exp. Res..

[4] Go Y.M., Jones D.P. (2013). Thiol/disulfide redox states in signaling and sensing. Crit. Rev. Biochem. Mol. Biol..

[5] Saurin A.T., Neubert H., Brennan J.P., Eaton P. (2004). Widespread sulfenic acid formation in tissues in response to hydrogen peroxide. Proc. Natl. Acad. Sci. U. S. A..

[6] James A.M., Hoogewijs K., Logan A., Hall A.R., Ding S., Fearnley I.M., Murphy M.P. (2017). Non-enzymatic N-acetylation of lysine residues by AcetylCoA often occurs via a proximal S-acetylated thiol intermediate sensitive to glyoxalase II. Cell Rep..

[7] Adoni K.R., Cunningham D.L., Heath J.K., Leney A.C. (2022). FAIMS enhances the detection of PTM crosstalk sites. J. Proteome Res..

[8] Leutert M., Entwisle S.W., Villen J. (2021). Decoding post-translational modification crosstalk with proteomics. Mol. Cell. Proteomics.

[9] Requena J.R., Fu M.X., Ahmed M.U., Jenkins A.J., Lyons T.J., Thorpe S.R. (1996). Lipoxidation products as biomarkers of oxidative damage to proteins during lipid peroxidation reactions. Nephrol. Dial. Transplant..

[10] Zhong H., Yin H. (2015). Role of lipid peroxidation derived 4-hydroxynonenal (4-HNE) in cancer: focusing on mitochondria. Redox Biol..

[11] Perry E.A., Castellani R.J., Moreira P.I., Nunomura A., Lui Q., Harris P.L., Sayre L.M., Szweda P.A., Szweda L.I., Zhu X. (2013). Neurofilaments are the major neuronal target of hydroxynonenal-mediated protein cross-links. Free Radic. Res..

[12] Xu G., Liu Y., Sayre L.M. (2000). Polyclonal antibodies to a fluorescent 4-hydroxy-2-nonenal (HNE)-derived lysine-lysine cross-link: characterization and application to HNE-treated protein and in vitro oxidized low-density lipoprotein. Chem. Res. Toxicol..

[13] Ren Y., Li J., Dai X. (2026). Reactive oxygen species in health and disease. Mol Biomed.

[14] Ali I., Conrad R.J., Verdin E., Ott M. (2018). Lysine acetylation goes global: from epigenetics to metabolism and therapeutics. Chem. Rev..

[15] Carrico C., Cruz A., Walter M., Meyer J., Wehrfritz C., Shah S., Wei L., Schilling B., Verdin E. (2023). Coenzyme A binding sites induce proximal acylation across protein families. Sci. Rep..

[16] Ago T., Liu T., Zhai P., Chen W., Li H., Molkentin J.D., Vatner S.F., Sadoshima J. (2008). A redox-dependent pathway for regulating class II HDACs and cardiac hypertrophy. Cell.

[17] Jansch N., Meyners C., Muth M., Kopranovic A., Witt O., Oehme I., Meyer-Almes F.J. (2019). The enzyme activity of histone deacetylase 8 is modulated by a redox-switch. Redox Biol..

[18] Yang J., Gupta V., Carroll K.S., Liebler D.C. (2014). Site-specific mapping and quantification of protein S-sulphenylation in cells. Nat. Commun..

[19] Shen L.F., Chen Y.J., Liu K.M., Haddad A.N.S., Song I.W., Roan H.Y., Chen L.Y., Yen J.J.Y., Chen Y.J., Wu J.Y. (2017). Role of S-Palmitoylation by ZDHHC13 in mitochondrial function and Metabolism in Liver. Sci. Rep..

[20] Keenan E.K., Bareja A., Lam Y., Grimsrud P.A., Hirschey M.D. (2024). Cysteine S-acetylation is a post-translational modification involved in metabolic regulation. bioRxiv.

[21] Lia A., Dowle A., Taylor C., Santino A., Roversi P. (2020). Partial catalytic Cys oxidation of human GAPDH to Cys-sulfonic acid. Wellcome Open Res..

[22] Tsuchiya Y., Peak-Chew S.Y., Newell C., Miller-Aidoo S., Mangal S., Zhyvoloup A., Bakovic J., Malanchuk O., Pereira G.C., Kotiadis V. (2017). Protein CoAlation: a redox-regulated protein modification by coenzyme A in mammalian cells. Biochem. J..

[23] Brett C., Gout I. (2025). The two faces of coenzyme A in cellular biology. Free Radic. Biol. Med..

[24] Reyes J.S., Fuentes-Lemus E., Fierro A., Rivero-Rodriguez K., Arenas F., Davies M.J., Lopez-Alarcon C. (2025). Inactivation of human glucose 6-phosphate dehydrogenase (G6PDH) by peroxyl radicals is strongly modulated by its substrate and cofactor. Free Radic. Biol. Med..

[25] James A.M., Smith A.C., Smith C.L., Robinson A.J., Murphy M.P. (2018). Proximal cysteines that enhance Lysine N-Acetylation of cytosolic proteins in mice are less conserved in longer-living species. Cell Rep..

[26] McGinnis C.D., Harris P.S., Graham B.I.M., Marentette J.O., Michel C.R., Saba L.M., Reisdorph R., Roede J.R., Fritz K.S. (2025). Acetylation of proximal cysteine-lysine pairs by alcohol metabolism. Redox Biol..

[27] Gould N.S., Evans P., Martinez-Acedo P., Marino S.M., Gladyshev V.N., Carroll K.S., Ischiropoulos H. (2015). Site-specific proteomic mapping identifies selectively modified regulatory cysteine residues in functionally distinct protein networks. Chem. Biol..

[28] Pehar M., Ball L.E., Sharma D.R., Harlan B.A., Comte-Walters S., Neely B.A., Vargas M.R. (2016). Changes in protein expression and lysine acetylation induced by decreased glutathione levels in astrocytes. Mol. Cell. Proteomics.

[29] Rabe von Pappenheim F., Wensien M., Ye J., Uranga J., Irisarri I., de Vries J., Funk L.M., Mata R.A., Tittmann K. (2022). Widespread occurrence of covalent lysine-cysteine redox switches in proteins. Nat. Chem. Biol..

[30] Dinkova-Kostova A.T., Holtzclaw W.D., Cole R.N., Itoh K., Wakabayashi N., Katoh Y., Yamamoto M., Talalay P. (2002). Direct evidence that sulfhydryl groups of Keap1 are the sensors regulating induction of phase 2 enzymes that protect against carcinogens and oxidants. Proc. Natl. Acad. Sci. U. S. A..

[31] Mertins P., Qiao J.W., Patel J., Udeshi N.D., Clauser K.R., Mani D.R., Burgess M.W., Gillette M.A., Jaffe J.D., Carr S.A. (2013). Integrated proteomic analysis of post-translational modifications by serial enrichment. Nat. Methods.

[32] Weinert B.T., Scholz C., Wagner S.A., Iesmantavicius V., Su D., Daniel J.A., Choudhary C. (2013). Lysine succinylation is a frequently occurring modification in prokaryotes and eukaryotes and extensively overlaps with acetylation. Cell Rep..

[33] Ibrahim L., Stanton C., Nutsch K., Nguyen T., Li-Ma C., Ko Y., Lander G.C., Wiseman R.L., Bollong M.J. (2023). Succinylation of a KEAP1 sensor lysine promotes NRF2 activation. Cell Chem. Biol..

[34] Marino S.M., Gladyshev V.N. (2012). Analysis and functional prediction of reactive cysteine residues. J. Biol. Chem..

[35] Chen H.R., Sun Y., Mittler G., Rumpf T., Shvedunova M., Grosschedl R., Akhtar A. (2024). MOF-mediated PRDX1 acetylation regulates inflammatory macrophage activation. Cell Rep..

[36] Parmigiani R.B., Xu W.S., Venta-Perez G., Erdjument-Bromage H., Yaneva M., Tempst P., Marks P.A. (2008). HDAC6 is a specific deacetylase of peroxiredoxins and is involved in redox regulation. Proc. Natl. Acad. Sci. U. S. A..

[37] Carroll B., Otten E.G., Manni D., Stefanatos R., Menzies F.M., Smith G.R., Jurk D., Kenneth N., Wilkinson S., Passos J.F. (2018). Oxidation of SQSTM1/p62 mediates the link between redox state and protein homeostasis. Nat. Commun..

[38] You Z., Jiang W.X., Qin L.Y., Gong Z., Wan W., Li J., Wang Y., Zhang H., Peng C., Zhou T. (2019). Requirement for p62 acetylation in the aggregation of ubiquitylated proteins under nutrient stress. Nat. Commun..

[39] Li M., Xiong J., Yang L., Huang J., Zhang Y., Liu M., Wang L., Ji J., Zhao Y., Zhu W.G. (2022). Acetylation of p62 regulates base excision repair through interaction with APE1. Cell Rep..

[40] Hennig P., Fenini G., Di Filippo M., Karakaya T., Beer H.D. (2021). The pathways underlying the multiple roles of p62 in inflammation and cancer. Biomedicines.

[41] Huang X., Yao J., Liu L., Chen J., Mei L., Huangfu J., Luo D., Wang X., Lin C., Chen X. (2023). S-acylation of p62 promotes p62 droplet recruitment into autophagosomes in mammalian autophagy. Mol. Cell.

[42] Skou L.D., Johansen S.K., Okarmus J., Meyer M. (2024). Pathogenesis of DJ-1/PARK7-Mediated Parkinson's disease. Cells.

[43] Choi J., Tak S., Jung H.M., Cha S., Hwang E., Lee D., Lee J.H., Ryu K.S., Park C. (2023). Kinetic evidence in favor of glyoxalase III and against deglycase activity of DJ-1. Protein Sci..

[44] Coukos J.S., Lee C.W., Pillai K.S., Shah H., Moellering R.E. (2023). PARK7 catalyzes stereospecific detoxification of Methylglyoxal consistent with glyoxalase and not deglycase function. Biochemistry.

[45] Gao Q., Jacob-Dolan J.W., Scheck R.A. (2023). Parkinsonism-associated protein DJ-1 is an antagonist, not an eraser, for protein glycation. Biochemistry.

[46] Witt A.C., Lakshminarasimhan M., Remington B.C., Hasim S., Pozharski E., Wilson M.A. (2008). Cysteine pKa depression by a protonated glutamic acid in human DJ-1. Biochemistry.

[47] Blackinton J., Lakshminarasimhan M., Thomas K.J., Ahmad R., Greggio E., Raza A.S., Cookson M.R., Wilson M.A. (2009). Formation of a stabilized cysteine sulfinic acid is critical for the mitochondrial function of the parkinsonism protein DJ-1. J. Biol. Chem..

[48] Im J.Y., Lee K.W., Junn E., Mouradian M.M. (2010). DJ-1 protects against oxidative damage by regulating the thioredoxin/ASK1 complex. Neurosci. Res..

[49] Waak J., Weber S.S., Gorner K., Schall C., Ichijo H., Stehle T., Kahle P.J. (2009). Oxidizable residues mediating protein stability and cytoprotective interaction of DJ-1 with apoptosis signal-regulating kinase 1. J. Biol. Chem..

[50] Ito G., Ariga H., Nakagawa Y., Iwatsubo T. (2006). Roles of distinct cysteine residues in S-nitrosylation and dimerization of DJ-1. Biochem. Biophys. Res. Commun..

[51] Repici M., Straatman K.R., Balduccio N., Enguita F.J., Outeiro T.F., Giorgini F. (2013). Parkinson's disease-associated mutations in DJ-1 modulate its dimerization in living cells. J. Mol. Med. (Berl.).

[52] Cohen T.J., Hwang A.W., Unger T., Trojanowski J.Q., Lee V.M. (2012). Redox signalling directly regulates TDP-43 via cysteine oxidation and disulphide cross-linking. EMBO J..

[53] Cohen T.J., Hwang A.W., Restrepo C.R., Yuan C.X., Trojanowski J.Q., Lee V.M. (2015). An acetylation switch controls TDP-43 function and aggregation propensity. Nat. Commun..

[54] Fazal R., Boeynaems S., Swijsen A., De Decker M., Fumagalli L., Moisse M., Vanneste J., Guo W., Boon R., Vercruysse T. (2021). HDAC6 inhibition restores TDP-43 pathology and axonal transport defects in human motor neurons with TARDBP mutations. EMBO J..

[55] Barrow E.R., Valionyte E., Baxter C.R., Yang Y., Herath S., O'Connell W.A., Lopatecka J., Strachan A., Woznica W., Stephenson H.N. (2024). Discovery of SQSTM1/p62-dependent P-bodies that regulate the NLRP3 inflammasome. Cell Rep..

[56] Eftekhari A., Sabir U., Kasumov T. (2025). The role of lysine acetylation in metabolic sensing and proteostasis. Pharmacol. Ther..

[57] Lorenzen I., Mullen L., Bekeschus S., Hanschmann E.M. (2017). Redox regulation of inflammatory processes is enzymatically controlled. Oxid. Med. Cell. Longev..

[58] Yanase T., Yashiro T., Takitani K., Kato S., Taniguchi S., Takayanagi R., Nawata H. (1997). Differential expression of PPAR gamma1 and gamma2 isoforms in human adipose tissue. Biochem. Biophys. Res. Commun..

[59] Liang H., Tang T., Huang H., Li T., Gao C., Han Y., Yuan B., Gao S., Wang H., Zhou M.L. (2022). Peroxisome proliferator-activated receptor-gamma ameliorates neuronal ferroptosis after traumatic brain injury in mice by inhibiting cyclooxygenase-2. Exp. Neurol..

[60] Cai J., Shi X., Wang H., Fan J., Feng Y., Lin X., Yang J., Cui Q., Tang C., Xu G. (2016). Cystathionine gamma lyase-hydrogen sulfide increases peroxisome proliferator-activated receptor gamma activity by sulfhydration at C139 site thereby promoting glucose uptake and lipid storage in adipocytes. Biochim. Biophys. Acta.

[61] Tian L., Wang C., Hagen F.K., Gormley M., Addya S., Soccio R., Casimiro M.C., Zhou J., Powell M.J., Xu P. (2014). Acetylation-defective mutant of ppargamma is associated with decreased lipid synthesis in breast cancer cells. Oncotarget.

[62] Kwak M.S., Kim H.S., Lkhamsuren K., Kim Y.H., Han M.G., Shin J.M., Park I.H., Rhee W.J., Lee S.K., Rhee S.G. (2019). Peroxiredoxin-mediated disulfide bond formation is required for nucleocytoplasmic translocation and secretion of HMGB1 in response to inflammatory stimuli. Redox Biol..

[63] Yang H., Antoine D.J., Andersson U., Tracey K.J. (2013). The many faces of HMGB1: molecular structure-functional activity in inflammation, apoptosis, and chemotaxis. J. Leukoc. Biol..

[64] Meng X., Na R., Peng X., Li H., Ouyang W., Zhou W., You X., Li Y., Pu X., Zhang K. (2024). Musashi-2 potentiates colorectal cancer immune infiltration by regulating the post-translational modifications of HMGB1 to promote DCs maturation and migration. Cell Commun. Signal..

[65] Lu B., Antoine D.J., Kwan K., Lundback P., Wahamaa H., Schierbeck H., Robinson M., Van Zoelen M.A., Yang H., Li J. (2014). JAK/STAT1 signaling promotes HMGB1 hyperacetylation and nuclear translocation. Proc. Natl. Acad. Sci. U. S. A..

[66] Wei L., Zhang W., Li Y., Zhai J. (2022). The SIRT1-HMGB1 axis: therapeutic potential to ameliorate inflammatory responses and tumor occurrence. Front. Cell Dev. Biol..

[67] Mu S., Li Z., Lin L., Wang D., Yang F., Chen L., Xian L., Lin K., Lin Y., Ye D. (2024). SIRT1-Mediated HMGB1 deacetylation suppresses neutrophil extracellular traps related to blood-brain barrier impairment after cerebral venous thrombosis. Mol. Neurobiol..

[68] Xu S., Zeng Z., Zhao M., Huang Q., Gao Y., Dai X., Lu J., Huang W., Zhao K. (2019). Evidence for SIRT1 mediated HMGB1 release from kidney cells in the early stages of hemorrhagic shock. Front. Physiol..

[69] Venereau E., Casalgrandi M., Schiraldi M., Antoine D.J., Cattaneo A., De Marchis F., Liu J., Antonelli A., Preti A., Raeli L. (2012). Mutually exclusive redox forms of HMGB1 promote cell recruitment or proinflammatory cytokine release. J. Exp. Med..

[70] Shi Y., Carroll K.S. (2020). Activity-based sensing for site-specific proteomic analysis of cysteine oxidation. Acc. Chem. Res..

[71] Harris P.S., McGinnis C.D., Michel C.R., Marentette J.O., Reisdorph R., Roede J.R., Fritz K.S. (2023). Click chemistry-based thiol redox proteomics reveals significant cysteine reduction induced by chronic ethanol consumption. Redox Biol..

[72] Soens R.W., Anderson B.J., Lancaster N.M., Kumar M., Hanssen J.K., Galmozzi A., Grant T., Overmyer K.A., Coon J.J. (2026). Atlas of lysine acetylation in the mouse. bioRxiv.

[73] Abramson J., Adler J., Dunger J., Evans R., Green T., Pritzel A., Ronneberger O., Willmore L., Ballard A.J., Bambrick J. (2024). Accurate structure prediction of biomolecular interactions with AlphaFold 3. Nature.

[74] Pakhrin S.C., Pokharel S., Pratyush P., Chaudhari M., Ismail H.D., Kc D.B. (2023). LMPhosSite: a deep learning-based approach for general protein phosphorylation site prediction using embeddings from the local window sequence and pretrained protein language model. J. Proteome Res..

[75] Pokharel S., Pratyush P., Heinzinger M., Newman R.H., Kc D.B. (2022). Improving protein succinylation sites prediction using embeddings from protein language model. Sci. Rep..

[76] Meng L., Chen X., Cheng K., Chen N., Zheng Z., Wang F., Sun H., Wong K.C. (2024). TransPTM: a transformer-based model for non-histone acetylation site prediction. Briefings Bioinf..

[77] Kennedy P.H., Alborzian Deh Sheikh A., Balakar M., Jones A.C., Olive M.E., Hegde M., Matias M.I., Pirete N., Burt R., Levy J. (2024). Post-translational modification-centric base editor screens to assess phosphorylation site functionality in high throughput. Nat. Methods.

[78] Reimer B.M., Awoonor-Williams E., Golosov A.A., Hornak V. (2025). CovCysPredictor: predicting selective covalently modifiable cysteines using protein structure and interpretable machine learning. J. Chem. Inf. Model..

[79] Erdos G., Dosztanyi Z. (2024). AIUPred: combining energy estimation with deep learning for the enhanced prediction of protein disorder. Nucleic Acids Res..

